# Comparing In-Hospital Mortality Prediction by Senior Emergency Resident's Judgment and Prognostic Models in the Emergency Department

**DOI:** 10.1155/2023/6042762

**Published:** 2023-05-15

**Authors:** Zahra Rahmatinejad, Samira Peiravi, Benyamin Hoseini, Fatemeh Rahmatinejad, Saeid Eslami, Ameen Abu-Hanna, Hamidreza Reihani

**Affiliations:** ^1^Department of Medical Informatics, Faculty of Medicine, Mashhad University of Medical Sciences, Mashhad, Iran; ^2^Department of Emergency Medicine, Faculty of Medicine, Mashhad University of Medical Sciences, Mashhad, Iran; ^3^Pharmaceutical Research Center, Mashhad University of Medical Sciences, Mashhad, Iran; ^4^Department of Health Information Technology, Faculty of Paramedical Sciences, Mashhad University of Medical Sciences, Mashhad, Iran; ^5^Department of Medical Informatics, Amsterdam UMC Location University of Amsterdam, Netherlands

## Abstract

**Background:**

A comparison of emergency residents' judgments and two derivatives of the Sequential Organ Failure Assessment (SOFA), namely, the mSOFA and the qSOFA, was conducted to determine the accuracy of predicting in-hospital mortality among critically ill patients in the emergency department (ED).

**Methods:**

A prospective cohort research was performed on patients over 18 years of age presented to the ED. We used logistic regression to develop a model for predicting in-hospital mortality by using qSOFA, mSOFA, and residents' judgment scores. We compared the accuracy of prognostic models and residents' judgment in terms of the overall accuracy of the predicted probabilities (Brier score), discrimination (area under the ROC curve), and calibration (calibration graph). Analyses were carried out using R software version R-4.2.0.

**Results:**

In the study, 2,205 patients with median age of 64 (IQR: 50-77) years were included. There were no significant differences between the qSOFA (AUC 0.70; 95% CI: 0.67-0.73) and physician's judgment (AUC 0.68; 0.65-0.71). Despite this, the discrimination of mSOFA (AUC 0.74; 0.71-0.77) was significantly higher than that of the qSOFA and residents' judgments. Additionally, the AUC-PR of mSOFA, qSOFA, and emergency resident's judgments was 0.45 (0.43-0.47), 0.38 (0.36-0.40), and 0.35 (0.33-0.37), respectively. The mSOFA appears stronger in terms of overall performance: 0.13 vs. 0.14 and 0.15. All three models showed good calibration.

**Conclusion:**

The performance of emergency residents' judgment and the qSOFA was the same in predicting in-hospital mortality. However, the mSOFA predicted better-calibrated mortality risk. Large-scale studies should be conducted to determine the utility of these models.

## 1. Introduction

The emergency department (ED) is the doorway for patients with acute illnesses, and physicians have an essential role in this setting, who make decisions about admission, discharge, and resource allocation [1–3]. Patients presenting to the ED with deteriorating vital signs are typically in critical condition and need immediate treatment [4–6]. It is common for physicians to be faced with ambiguous and stressful situations on a regular basis [7, 8]. Their main duties include identifying patients at high risk of mortality, estimating their severity of illness, determining their prognosis, and selecting the appropriate interventions [9–12].

Suitable assessment in the ED is integral for prioritizing critically ill patients, timely management of accurate diagnostic and therapeutic interventions, and optimal resource utilization [13]. One of the potentially useful tools in such a crucial situation would be scoring systems which are quantitative methods for reinforcing clinical judgment [14, 15]. Predictive models are not usually addressed as a critical component of treatment, but they can be crucial for improving clinical decisions [15–17]. There are many different scoring systems available. However, we considered factors of the ED including feasibility and practicality. In this work, we considered the modified Sequential Organ Failure Assessment (mSOFA) and quick Sepsis-Related Organ Failure Assessment (qSOFA), which are mainly based on clinical variables, and assessing the coagulation system by measuring platelets and renal function by creatinine [18, 19] (see Table 1). As a further reason for choosing mSOFA and qSOFA for comparison, residents were only asked to express their judgment after their first visit, before requesting any additional investigations (including lab results and CT scans). Their information is almost like the information obtained from the variables used in the models. We can include APACHE II, SAPS II, and MPM as scoring systems that have been compared to physicians' judgment, but these systems are all related to the ICU environment [20–22]. Besides, those models applied in EDs were examined only on specific diseases such as sepsis, pulmonary embolism, and shock [23–25]. It should be noted; although the mSOFA and qSOFA prediction models were initially developed to predict the mortality of sepsis patients, many studies have evaluated these systems on patients with other diseases such as pneumonia or nontraumatic disorders [26, 27]. Therefore, the purpose of this study is to compare the prognostic ability of residents' judgment with two SOFA derivative models, one with only clinical variables (qSOFA) and the other with some additional laboratory information (mSOFA) for in-hospital mortality among all cases. The physicians in this study were senior residents in the 3rd year of their residency in a 3-year emergency medicine residency program. On the other hand, we were interested in knowing our residents' competency in discrimination of ill patients in their first contact with ED patients and comparing it with a measurable tool. It is worth mentioning that physician judgment is utilized in their first visit after triaging the patients by the nurse.

## 2. Method

### 2.1. Study Design, Setting, and Participants

This prospective cohort study was conducted between March 2016 and March 2017 on 3,064 patients presented to the ED in Imam Reza Hospital, Iran. This setting has over 200,000 patient visits annually and is one of the referral centers where patients are referred to. The research committee at Mashhad University of Medical Sciences approved this investigation with a waiver of informed consent, under number: 1395.166, according to the Declaration of Helsinki principles. The reason to waive the informed consent was that the researchers were not in direct contact with patients and only routine clinical data collected through the hospital information system were achieved. Moreover, patients were not exposed to any interventions, such as special treatment or sampling, and the data were analyzed anonymously.

According to their acuity and resource needs, the Emergency Severity Index (ESI) classifies patients into five clinically relevant groups based on the Emergency Severity Index. The patients in level 1 are the most critical, while the patients in level 5 are the least severely ill patients, and they can walk. Generally, the patients in levels 1 to 3 need ED or hospital admission.

### 2.2. Inclusion and Exclusion Criteria

**Figure 1:**
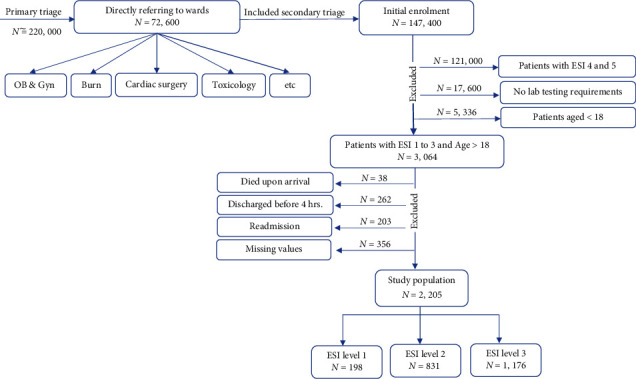
An overview of the enrollment process for the study population.

We included only adult patients (>18 years old) who were assigned to triage acuity levels from 1 to 3 (according to the ESI of 1 to 3) in the ED. The patients were excluded if (1) died upon arrival, (2) returned to ED with the same diagnosis, (3) were discharged before four hours, or (4) were directly referred to the particular departments for burns, trauma, poisoning, obstetrics, and surgery (this center applied two-stage triage). Our models do not apply to these groups of patients. In addition, we excluded instances with missing values from the dataset when calculating the prediction score was impossible due to at least one assessment missing (Figure 1).

### 2.3. Calculation of the Risk Score System

Table Error! Reference source not found. displays vital components of mSOFA and qSOFA measured upon patient arrival in the ED. We also collected demographic data, triage level, and physicians' clinical assessment for each patient. In-hospital mortality is defined as an endpoint for evaluating the performance of the mSOFA and qSOFA scores and physician's clinical assessment among patients presenting to ED. After determining the level of triage by the nurse, the patients were examined by one of five senior emergency residents who were part of the patient treatment. Following the resident's first visit, we asked them to make clinical judgments solely based on medical history and physical examination (without lab results and CT scans). They assessed the likelihood of in-hospital mortality for each patient based on vital signs such as body temperature, heart rate, respiratory rate, blood pressure, oxygen saturation, history, and clinical examination including the level of patient consciousness. The base of clinical judgment was almost like the information obtained from the variables used in the models. This score was scaled between 0 and 100 (alive, 0; deceased, 100) [13, 28]. We converted quantitative values into three categories to simplify the comparisons of the prognostic ability of the residents' judgment with two SOFA derivative models [29–31] (see also Table 2). During the study period, all residents were blinded to the outcomes.

### 2.4. Performance Assessment and Comparison

The predictive performance of the qSOFA, mSOFA, and physician's clinical judgment was evaluated using the area under the receiver-operating characteristic (AUROC) curve to measure the model's discriminatory ability. The AUC value of 1 indicated perfect discrimination, while the value of 0.5 indicated no discrimination. The AUCs were compared using the DeLong method [32]. The accuracy of the predicted probability was measured by the Brier score (BS), which is a measure of error with a value of 0 as the perfect accuracy.

Logistic regression is a robust method to estimate the probability of a binary-dependent variable (in our case in-hospital mortality) based on independent variables (such as blood pressure and temperature). We applied logistic regression to obtain two models to predict the probability of in-hospital mortality, one using the qSOFA and the other using mSOFA. The physicians' prognoses had been already expressed as probabilities. We used 1000 replicate bootstrap datasets to measure the bias-corrected estimate of the AUC of the three sets of predictions (from qSOFA, mSOFA, and the physicians' predictions). We reported the mean AUROC with 95% confidence intervals (CI) for each model (two logistic regression models and the physicians' predictions). Additionally, each model's Precision-Recall AUC (AUC-PR) was performed, which shows the balance between the positive predictive value and the sensitivity.

We also evaluated these models based on the overall accuracy of the predicted probabilities by the Brier score and calibration with calibration graphs [33]. To get insight into the (mis)calibration of the physicians' judgments, we regressed the outcome on the log odds of their predictions.

We summarized data as the relative frequency (%) for categorical variables and as median and interquartile ranges (IQR) for continuous variables. The Mann–Whitney *U* test was applied for comparing continuous variables and the chi-square test or Fisher's exact test for categorical variables. A *P* value < 0.05 indicated statistical significance. Analyses were carried out using R software version R-4.2.0.

## 3. Results

In total, we enrolled 2,205 patients during one year. Of these, 53% were categorized into ESI level III, 38% in ESI level II, and 9% in ESI level I. The median age was 64 (IQR: 50-77) years, survivors' median age was 63 (48-76) years, while nonsurvivors' median age was 70 (57-80) years. Out of the 2,205 patients, 1029 (46.7%) were female, and 1176 (53%) were male.

According to the International Classification of Diseases (ICD-10), the frequent diagnoses were related to the digestive system (549, 24.9%), neoplasms (352, 16%), the respiratory system (296, 13.4%), circulatory system (279, 12.7%), and urinary system (235, 10.7%), and the rest of the diseases were related to other systems (387, 13%). About 19.3% of the patients (426 out of 2205) passed away during staying in hospital. Among all assessed patients, those who suffered from cancer had the highest mortality rate (21%). The highest in-hospital mortality was observed among patients with ESI level I (38.8%).

Table 2 summarizes the patients' characteristics stratified by in-hospital mortality, which was 19%. Those who were deceased were older and had significantly higher qSOFA, mSOFA, and physician prognosis scores.

The median mSOFA, qSOFA, and physician prognosis scores were 2 (IQR: 0-3), 0 (IQR: 0-1), and 20 (IQR: 10-40), respectively, across the entire sample. A positive qSOFA score (score ≥ 2) corresponded to 10% of survivors and 34% of nonsurvivors. The linear predictor of the logistic regression model for mSOFA and qSOFA were as follows:

*LP*_*mSOFA*_ = −2.56 + 7.94 × *mSOFA*, 

LPqSOFA = −2.25 + 2.9868 × qSOFA.

The probability of death was obtained from the linear predictor, LP, by 1/(1 + exp(LP)). When regressing the outcome on the log odds (LO) of the physician's predicted probability, we obtain the following linear predictor: = −0.725 + 0.652 × LO.

**Figure 2:**
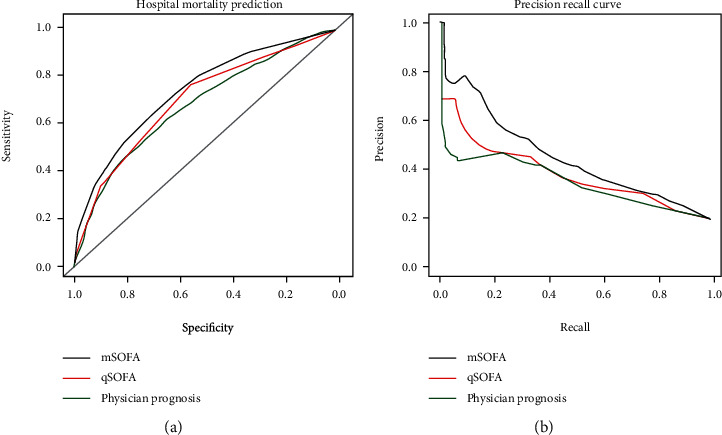
AUC-ROC (a) and AUC-PR (b) for the three models evaluated. In both cases, a higher area under the curve indicates better predictions. The mSOFA model performs better than other models.

The AUROC of the mSOFA, qSOFA, and physician prognosis is 0.74 (0.71-0.77), 0.70 (0.67-0.73), and 0.68 (0.65-0.71), respectively (Figure 2). Generally, the performance of mSOFA was better than that of the other models. Further performance indices are presented in Table 3. Based on the calibration graphs, the actual mortality is comparable to the prediction models (qSOFA and mSOFA). The prognoses of physicians in the third graph without recalibration, based on regressing the outcome on the predictions, indicate overpredictions between 15 and 25% and underpredictions over 30%.

## 4. Discussion

### 4.1. Main Finding

This study compared the prognostic performance of physicians' clinical judgments with two derivative versions of the SOFA scoring system (mSOFA and qSOFA) in terms of in-hospital mortality. The AUROC of the mSOFA was significantly better than that of the qSOFA II (*P* < 0.001) and physicians' prognosis (*P* < 0.001). The AUROC difference between the physicians' prognosis and qSOFA was not statistically significant (*P* = 0.20) (Figure 2). In our study, we demonstrated that the mSOFA model can accurately predict in-hospital mortality as this model obtained the highest prognostic accuracy. Additionally, the model based on the mSOFA score was associated with the lowest prediction errors as compared to the actual outcomes. The qSOFA demonstrated the highest sensitivity (0.76), but the lowest specificity (0.57) for the in-hospital mortality endpoint, followed by mSOFA and the physicians' prognosis predictions. The specificity of mSOFA model was 12 and 6 percentage points higher than that of the qSOFA and the physicians' predictions, respectively. Having a low specificity might lead to false high-risk alerts and consequently can cause overuse of the resources, while having a low sensitivity might lead to missing critically ill patients and, as a result, increase mortality and morbidity. Generally, the challenge of combining high specificity and sensitivity in a screening tool is well-known [34].

**Figure 3:**
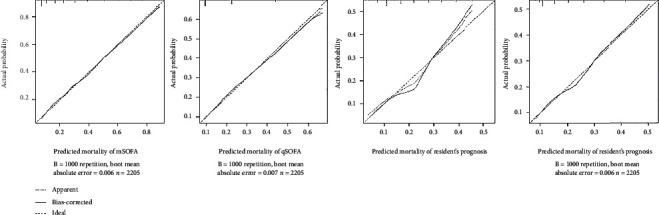
The calibration graphs reveal that the actual mortality is similar to the predicted one by the prediction models (qSOFA, mSOFA). Without recalibration by regressing the outcome on the predictions, physicians' prognoses indicate overpredictions of 15 to 25% and underpredictions above 30%. After recalibration, in the fourth graph, the calibration is improved.

The calibration curves demonstrate an agreement between observed and predicted probabilities for the models but worse calibration for the physicians (the third graph in Figure 3): the physicians' prognosis tended to overestimate the probability of death in the midrange and underestimate the probability in the high. Recalibrating their predictions, the calibration improved. However, note that in clinical practice and contrast to the objective prediction models, recalibration of the physicians' predictions is not realistic. Also, after recalibrating the physician's predictions, the linear predictor had an intercept of -0.725 and a slope of 0.652, which are far from their ideal values for perfect calibration of 0 and 1, respectively.

We note that the physicians are requested to estimate the patients' in-hospital mortality immediately after the presentation so that it can be affected by the triage level set at the patient's arrival, the doctor's fear of legal issues, excessive concern of companions, the instability of the patient at the time of referral, or the lack of knowledge of the patient's test results.

Accurate judgment about the patient, apart from the patient's main complaint or the history sometimes presented by the unconscious patient's uninformed companions, depends on the expertise of the physician in physical examination, the physician's attention to the patient's signs and symptoms, and the physician's review of organ systems as a part of a whole (including the respiratory, cardiovascular, nervous, coagulation, hepatic, and renal systems).

The fact that mSOFA differentiated between survivors and nonsurvivors is better than qSOFA and the physicians' prognosis (0.74 vs. 0.70 and 0.68, respectively), and the improved calibration demonstrated the potential merit of using prognostic models to improve, and not to replace, the physician's perception. Providing these models to physicians can help them better estimate patients' prognoses. They can augment the physician's clinical judgment—especially in overcrowded situations of the ED. Scoring systems can be helpful to frequently and objectively assess a patient's condition over time for comparison with their previous condition, revealing recovery or deterioration in the patient's health. However, we suggest evaluating the cost-effectiveness of these scoring systems [35, 36].

### 4.2. Comparison to Other Similar Studies

Several studies have compared physicians' judgment with prognostic models for predicting outcomes, mainly in the ICU. Other similar studies have focused on a single disorder or a single model (see Table 4). To our knowledge, this is the first study to compare physicians' prognoses with scoring systems in the ED setting on a wide range of diseases. Additionally, the studies provided mixed evidence on whether physicians' judgments are more accurate than prognostic models or vice versa [37].

According to Chiew et al., the models without laboratory data resulted in remarkably reduced performance [42]. It is possible that the superiority of mSOFA is due to the fact that it addresses the laboratory parameters that may improve the model's performance, while physicians are unaware of such results when making their clinical judgment. However, physicians have extra knowledge not included in the model, such as past medical history and other cues when visiting the patient. The performance of the models evaluated in our study differed from the models evaluated in other countries for a variety of reasons. First, the original models were created for western people and are now being applied to an Iranian population. Second, the disparity in quality and standards of treatment and the technology utilized could be the other potential reasons.

Since clinical judgment is subjective, having objective scores can be helpful to physicians, especially those who are not adequately experienced. Our study highlighted that using scoring systems can help physicians, especially junior physicians (or physicians without experience), make more realistic prognoses, and in turn, improve patient management [24].

Another similar study, carried out on patients with community-acquired pneumonia (CAP), reported the results in line with our findings. Although the qSOFA outperformed SIRS and had more clinical usefulness as quick tools for patients with the CAP in the ED, the discriminatory power of mSOFA was still better than qSOFA [43]. In accordance with our findings, Ebrahimian et al. reported that the AUROCs of mSOFA predict serious complications more accurately than those of qSOFA in EDs (0.88 vs. 0.71), so this model is a suitable instrument for triaging nontraumatic patients [44]. There is another study which claimed that machine learning models outperformed the judgment of internal medicine physicians [41]. In our study, it was found that clinical judgment overpredicted mortality. It should be noted that in the recent literature reports, there was no difference in the discriminatory performance of PIRO, MEDS, and clinical judgement categories in the low-risk cohort for the prediction of 28-day mortality. Similarly, the evidence implied that there are no significant differences in performance between the model and physicians in predicting clinical deterioration at 24 hours. However, the combined algorithm using both models outperforms the individual models [13]. Another study reported that the qSOFA did not improve physician judgment or outperform it when predicting 28-day in-hospital mortality among infected ED patients. Additionally, a multivariate modeling approach which included qSOFA did not improve discrimination in mortality prediction [23]. In contrast, a study revealed that clinical judgement was a reliable method to stratify patients at either the ICU or the general ward admission in ED patients with sepsis, and the qSOFA scores did not add value to this stratification but performed better on the prediction of mortality [40]. Mortality in our study was in the midrange of the existing literature (see Table 4).

Generally, clinical researchers would benefit greatly from an index of clinical severity, especially for studies that is aimed at assessing the effectiveness or efficacy of therapeutic interventions. Using a reliable index, patients could be randomly assigned to groups based on their severity, eliminating any concern that the short-term risk of the two groups would not be truly balanced. As researchers and administrators progress forward in prioritizing severe clinical cases, allocating the ED and ICU beds, and distributing intensive care capacity, they will be encouraged and supported by applying prediction models for benchmarking purposes [45]. As a result of early identification, all critically ill patients benefit since the patient can be treated and monitored at an earlier stage [46].

### 4.3. Strengths and Limitations of the Current Study

This study was designed as a prospective study and included a wide range of ill patients. Moreover, we applied a comprehensive evaluation based on various performance measures. However, the exclusion criteria may limit the scope of the generalizability of the models, especially in terms of the excluded patient subpopulations. In addition, this is not a multicenter study. Furthermore, one should be aware that the diagnostic and treatment modalities ordered by the physicians are affected by the physicians' judgment. Thus, there may be a relationship between the physician's judgment and the outcome.

## 5. Conclusion

Generally, emergency residents' judgments had a predictive performance that resembled the performance of the qSOFA model in predicting in-hospital mortality in ED patients but worse than the mSOFA model. Further research is required to investigate the performance and accuracy of these models in large-scale reliability before using them in clinical practice. As a first step toward identifying high-risk patients and establishing a clinical decision process, a screening model can serve.

## Figures and Tables

**Table 1:** mSOFA and qSOFA scoring system.

Model (min-max)	Variables				
	Cardiovascular system	Respiratory system	Liver	Central nervous system	Renal
mSOFA (0-19)	**M** **A** **P**_(**mmHg**)_ < 70→1 Dopamine ≤ 5→2 Dobutamine any dose→2 Dopamine > 5→3 Epinephrine ≤ 0.1→3 Norepinephrine ≤ 0.1→3 Dopamine > 15→4 Epinephrine > 0.1→4 Norepinephrine > 0.1→4	**SpO2/FiO**_**2(mmHg)**_ >400→0 ≤400→1 ≤315→2 ≤235→3 ≤150→4	No scleral icterus→0 Jaundice→0 Scleral icterus→1 Jaundice→1	**GCS** 15→0 13–14→1 10–12→2 6–9→3 <6→4	**Creatinine**_**(mg/dL)**_ <1.2→0 1.2–1.9→1 2.0–3.4→2 3.5–4.9→3 >5.0→4

qSOFA (0-3)	**S** **B** **P**_(**mmHg**)_ ≤ 100→1	RR ≥ 22→1	NA	≤14→1	NA

Abbreviation: MAP: mean arterial pressure; RR: respiratory rate; CNS: central nervous system; SBP: systolic blood pressure; SpO2: oxygen saturation; FiO_2_: fraction of inspired oxygen; mSOFA: modified Sequential Organ Failure Assessment; qSOFA: quick Sepsis-Related Organ Failure Assessment.

**Table 2:** Baseline characteristics of study participants.

Characteristics	Nonsurvivors (*N* = 426)	Survivors (*N* = 1779)	Total (*N* = 2,205)	*P* value
Age (year)	70 (57-80)	63 (48-76)	64 (50-77)	<0.001^a^
*Gender*				
Male	194 (45.5%)	835 (46.9%)	1176 (53.3%)	0.62^b^
Female	232 (54.5%)	944 (53.1%)		

*mSOFA parameters*				
Icteric	26 (6%)	40 (2%)	185 (8%)	<0.001^b^
MAP (mmHg)	90 (74-102)	93 (83-103)	93.3 (82-103)	<0.001^a^
SpO_2_	95% (90-95)	95% (94-96)	95% (93-96)	<0.001^a^
FiO_2_	21% [21–40]	21% [21]	21% [21]	<0.001^a^
GCS	15 (13-15)	15 (15-15)	15 (15-15)	<0.001^a^
Creatinine	1.7 (1.1-2.9)	1.2 (0.9-1.8)	1.2 (0.9-2)	<0.001^a^
Platelets	215 (147-289)	180 (104-272)	210 (137-286)	0.007^a^

*qSOFA parameters*				
SBP	120 (100-139)	126 (110-140)	125 (110-140)	<0.001^a^
Respiratory rate	20 (18-25)	18 (17-20)	18 (17-21)	<0.001^a^
GCS	15 (13-15)	15 (15-15)	15 (15-15)	<0.001^a^

*Models*				
Physician prognosis	40 (20-50)	20 (10-30)	20 (10-40)	<0.001^a^
0-30	210 (49.3)	1348 (75.8)	1558 (70.7%)	
31-60	187 (43.9)	392 (22.1)	579 (26.3)	
≥61	29 (6.8)	38 (2.1)	67 (3%)	

qSOFA score	1 (1-2)	0 (0-1)	0 (0-1)	<0.001^a^
0	102 (23.9)	1009 (56.7)	1111 (50.4%)	
1	180 (42.3)	593 (33.3)	773 (35.1%)	
≥2	144 (33.8)	177 (9.9)	321 (14.6%)	

mSOFA score	4 (2-5)	0 (1-3)	2 (0-3)	<0.001^a^
0-4	286 (67.1)	1650 (92.7)	1936 (87.8%)	
5-8	116 (27.3)	121 (6.9)	237 (10.7%)	
≥9	24 (2.3)	8 (0.4)	32 (1.5%)	

*Triage level (ESI)*				
Level 1-resuscitation	77 (18.1%)	121 (6.8%)	198 (9%)	0.001^c^
Level 2-emergent	199 (46.7%)	632 (35.5%)	831 (37.7%)	
Level 3-urgent	150 (35.2%)	1026 (57.7%)	1176 (53.3%)	
Ventilation support	93 (21.8%)	30 (1.7%)	124 (5.6%)	<0.001^b^

*Diagnostic group based on ICD-10*				
Certain infectious and parasitic disease	59 (14%)	117 (7%)	176 (8%)	0.002^a^
Neoplasms, diseases of the blood	90 (21%)	193 (11%)	352 (16%)	
Diseases of the circulatory system	58 (14%)	221 (12%)	279 (12.7%)	
Diseases of the respiratory system	60 (14%)	236 (13%)	296 (13.4%)	
Diseases of the digestive system	79 (19%)	470 (26%)	549 (24.9%)	
Diseases of the urinary system	41 (10%)	194 (11%)	235 (10.7%)	
Other reasons	39 (9%)	348 (19%)	387 (17.5%)	

Abbreviation: MAP: mean arterial pressure; FiO_2_: fraction of inspired oxygen; MAP: mean arterial pressure; GCS: Glasgow Coma Scale. ^a^Analysis by independent-samples *t* test. ^b^Analysis by Fisher's exact test. ^c^Analysis by Chi-square test.

**Table 3:** Performance indices of the qSOFA, mSOFA, and physician prognosis models for predicting in-hospital mortality.

Models	AUC-ROC (95% CI)	AUC-PR (95% CI)	BS	Threshold	SE	SP	PPV	NPV	Acc
mSOFA	0.74 (0.71-0.77)	0.45 (0.43-0.47)	0.130	2.5	0.657	0.689	0.336	0.893	0.683
qSOFA	0.70 (0.67-0.73)	0.38 (0.36-0.40)	0.141	0.5	0.76	0.57	0.296	0.908	0.604
Physician prognosis	0.68 (0.65-0.71)	0.35 (0.33-0.37)	0.165	21	0.640	0.629	0.292	0.879	0.631

Abbreviations: AUROC: area under the receiver-operating characteristic curve; PRAUC: area under the precision-recall curve; CI: confidence interval; BS: Brier score; SE: sensitivity; SP: specificity; PPV: positive predictive value; NPV: negative predictive value; Acc: accuracy.

**Table 4:** Published studies comparing the performance of models with physician clinical judgment.

Study	Year	Country	Sample size (N)	Male gender (%)	Age^a^ Mean ± SD or median (IQR)	Outcome rate (%)	Setting	Diagnosis	Prognostic model	Outcome events	AUROC Model versus physician prognoses
[37]	1988	US	366	NA	NA	40%	ICU	Case-mix	APACHE II	Hospital mortality	0.89 vs. 0.89
[1]	1989	US	523	44%	56.6 ± 19.6	25%	ICU + Ward	Case-mix	APACHE II	Hospital mortality	0.83 vs. 0.89
[20]	1989	US	215	43%	57.9 ± 1.9: deceased 55.1 ± 2.4: survivors	30.2%	ICU	Case-mix	APACHE II	Hospital mortality	80 vs. 90
[38]	2010	US	137	NA	Neonate <4 weeks	34%	ICU	Candidiasis	Neonatal candidiasis	Hospital mortality	0.79 vs. 0.70
[13]	2019	US	1874	50%	56 (42–70)	1.9%	Internal medicine wards	Case-mix	EWS	Clinical deteriorations in 24 hours	0.73 vs. 0.70
[39]	2013	Netherlands	323	57%	66 ± 17	22%	ED	Sepsis	PIRO	28-day mortality	0.68 vs. 0.69 in high-risk 0.83 vs. 0.84 in low-risk
[40]	2017	Netherlands	193	56%	60 (48–71)	4.1%	ED	Sepsis	qSOFA PIRO	Hospital mortality	0.849 vs. 0.861 0.876 vs. 0.861
[23]	2020	US	405	60%	58.3 ± 16.5	8.2%	ED	Sepsis	qSOFA	28-day mortality	0.63 vs. 0.80
[10]	2019	US	314	57%	61.7 (17.1): deceased 55.6 (16.8): survivors	9.9%	ED	SIRS+	Biomarker	28-day mortality	0.72 vs. 0.78
[13]	2019	US	1874	51%	56 (42–70)	1.9%	Ward	Case-mix	EWS	24 h clinical deterioration	0.73 vs. 0.70
[21]	2004	Germany	412	59%	59 ± 19.5	17.7%	ICU	Case-mix	SAPS II	Hospital mortality	0.75 vs. 0.84
[22]	2001	US	235	54%	66 ± 17	18.85%	ICU	Non-trauma	MPM	24 h ICU mortality	NA
[24]	2018	Spain	154	37%	67 ± 18	8.4%	ED	Pulmonary embolism	PESI sPESI	30-day mortality	0.73 vs. 0.65 0.77 vs. 0.65
[25]	2018	China	220	62.73%	69.36 ± 15.88	13.6%	ED	Shock	LiPS	30-day mortality	0.72 vs. 0.62
[34]	2021	Sweden	323	58.5%	78 (72–85)	44.6%	ED	Sepsis	NEWS2, RETTS	Sepsis within 36 h from ED arrival	0.67 vs. 0.57 0.68 vs. 0.57
[41]	2021	UK	1,344	42%	70.8 (58.4-82.8)	13.0%	ED	Sepsis	Machine learning SOFA mREMS	31-day mortality	0.85 vs. 0.73 0.75 vs. 0.73 0.64 vs. 0.73
Current study	2020	Iran	2,205	53%	62 ± 18	19%	ED	Case-mix	mSOFA qSOFA	Hospital mortality	0.74 vs. 0.68 0.70 vs. 0.68

Abbreviation: APACHE II: Acute Physiology and Chronic Health Evaluation; qSOFA: quick Sepsis-Related Organ Failure Assessment; EWS: early warning score; SAPS: Simplified Acute Physiology Score; MPM: Mortality Prediction Model; PIRO: the predisposition, infection, response, and organ failure; PESI: Pulmonary Embolism Severity Index; sPESI: Simplified Pulmonary Embolism Severity Index; LiPS: Li's pragmatic shock; mSOFA: modified Sepsis-Related Organ Failure Assessment.

## Data Availability

Data analyzed in this study will be available upon reasonable request from the corresponding author.
